# Advanced paternal age is a risk factor for schizophrenia in Iranians

**DOI:** 10.1186/1744-859X-10-15

**Published:** 2011-04-24

**Authors:** Morteza Naserbakht, Hamid-Reza Ahmadkhaniha, Bahareh Mokri, Cassandra L Smith

**Affiliations:** 1 Social Medicine, Mental Health Research Center, Tehran University of Medical Sciences, Tehran, Iran; 2 Tehran University of Medical Science, Tehran, Iran; 3 Mental Health Research Center, Tehran University of Medical Sciences, Tehran, Iran; 4 Tehran Psychiatric Institute, Tehran, Iran; 5 Department of Internal Medicine, National Research Institute of Tuberculosis and Lung Disease (NRITLD), Masih Daneshvari Hospital, Shahid Beheshti University. M.C, Iran; 6 Biomedical Engineering Department, Boston University, Boston MA, USA

## Abstract

**Background:**

Since 1958 many, but not all studies have demonstrated that paternal age is a risk factor for schizophrenia. There may be many different explanations for differences between studies, including study design, sample size, collection criteria, heterogeneity and the confounding effects of environmental factors that can for example perturb epigenetic programming and lead to an increase in disease risk. The small number of children in Western families makes risk comparisons between siblings born at different paternal ages difficult. In contrast, more Eastern families have children both at early and later periods of life. In the present study, a cross-sectional population study in an Iranian population was performed to compare frequency of schizophrenia in younger offspring (that is, older paternal age) versus older offspring.

**Methods:**

A total of 220 patients with the diagnosis of schizophrenia (cases) from both psychiatric hospitals and private clinics and 220 individuals from other hospital wards (controls), matched for sex and age were recruited for this study. Patients with neurological problem, substance abuse, mental retardation and mood disorder were excluded from both groups.

**Results:**

Birth rank comparisons revealed that 35% vs 24% of the cases vs the controls were in the third or upper birth rank (*P *= 0.01). Also, the mean age of fathers at birth in case group (30 ± 6.26 years) was significantly more than the control group (26.45 ± 5.64 years; *P *= 0.0001). The age of 76 fathers at birth in case group was over 32 versus 33 fathers in control group. Individuals whose fathers' age was more than 32 (at birth) were at higher risk (2.77 times) for schizophrenia versus others (*P *< 0.0001, 95% CI 1.80 to 4.27). The maternal age at parturition of the case versus controls groups was 26.1 ± 5.41 vs 25.07 ± 4.47 (*P *= 0.02). Logistic regression analysis suggests that maternal age is less likely to be involved in the higher risk of schizophrenia than advanced parental age.

**Discussion:**

This study demonstrates a relationship between paternal age and schizophrenia in large families of an Iranian population. Arguments have been put forth that DNA bases changes or epigenetic changes in sperm account for the increased risk associated with older fathers. However, it would not be surprising that both *de novo *germline mutations and epigenetic changes contribute to disease occurrence because DNA replication and DNA methylation are closely linked at both the macromolecular level (that is, methylation closely follows replication), and at the metabolic level (both processes require folate), and susceptible to modulation by the environment. Further research on samples such as those collected here are needed to sort out the contributions of de novo mutations versus epigenetic changes to schizophrenia.

## Introduction

Schizophrenia is a chronic and disabling disorder with an approximate 1% incidence in the population, and 120 million afflicted individuals worldwide [1]. Hallucinations, delusions, and emotional, cognitive, and motor deficits are common symptoms that appear usually in late adolescence or early adulthood [2,3]. Many studies detected a genetic link to the disorder, although no single or small number of genes accounts for the majority of cases [4,5]. Systematic analyses of different pedigrees segregating schizophrenia suggested multiple genes with a complex inheritance pattern [6,7]. Child adoption studies have supported a polygenic and multifactorial aetiology [8].

Environmental factors linked to schizophrenia include winter parturition [9], maternal stress during pregnancy [10,11], second trimester and postnatal infection, labour and perinatal traumas, immigration, residency in urban areas and so on [12,13], and paternal occupation and age [14]. Paternal age has been linked to schizophrenia since 1958 [15]. Most studies including a recent meta-analysis have confirmed paternal age as a risk factor for schizophrenia [16-24], however negative results also exist in the literature [25]. The link between paternal age and schizophrenia, argues that *de novo *changes to DNA can lead to schizophrenia, because the sole biological contribution of fathers to progeny is DNA. *De novo *mutations are linked to neurodevelopmental disorders and cancers [26,27] and may explain the high prevalence of schizophrenia despite the decreased fertility of schizophrenia patient [14]

Malaspina suggested that single base pair mutations, trinucleotide repeat expansion, or genetic imprinting changes might be responsible for the observed effect [28]. However, DNA replication and epigenetic changes are closely linked at both the macromolecules level (for example, DNA replication and global DNA methylation), and the metabolic level (for example, synthesis of purines and deoxythymidine triphosphate (dTTP) and S-adenosyl methionine, the cofactor that donates a methyl group). Hence, it would not be surprising that paternal age influences both of these as well as other overlapping processes.

In 2002, Malaspina *et al*. reported that the paternal age at birth of sporadic cases averaged 4.7 years older than familial cases of schizophrenia, and attributed approximately 27% of the sporadic cases to paternal age, and likely due to *de novo *mutations [14]. However, Pulver *et al*. observed that paternal age is not different in (1) familial versus sporadic cases, and (2) first-degree and second-degree paternal relatives of probands, and argued that these results did not support the linkage of paternal age to spontaneous forms of schizophrenia [29]. It is quite clear that studies on large families and pre/post-index generations are important to distinguish genetic from sporadic cases. However, it should be noted that, if advanced paternal age is a risk factor for schizophrenia, the frequency and coincidence of disease would be more in children of fathers with higher age and could be misinterpreted as genetic inheritance. This may obscure the detection of sporadic versus familial cases that could be overcome in part by the examining the effects of higher birth rank, accompanied with advanced paternal age, on schizophrenia pathogenesis in large families.

This study examined the relationship between paternal age and schizophrenia in an Iranian population, a distinct cultural group not previously studied. Typical Iranian families are large with a wide variation in paternal age not typically seen in Western families. The study of large families provides us with an opportunity to examine the relationship between birth order, paternal age and schizophrenia. If advanced paternal age is an important risk factor for schizophrenia, disease frequency would be greater in children with higher birth rank as well (that is, higher paternal age). Hence, we examined the effects of higher birth rank and advanced paternal age on schizophrenia.

## Methods

This case-control study was performed in Tehran in 2005 and 2006 recruiting 220 patients (case group) with a diagnosis of schizophrenia from both psychiatric hospitals and private clinics. The diagnosis was made based on *Diagnostic and Statistical Manual of Mental Disorders*, fourth edition (DSM-IV) criteria in a semistructured interview by two psychiatrists. The control group was made up of 220 individuals from non-psychiatric hospital wards of the same district, matched for sex (*P *= 0.84, χ^2 ^test) and age (*P *= 0.37, t test) versus the case group. Individuals with any neurological problem, substance abuse, mental retardation or mood disorder were excluded from the case and control groups. Patients in an acute psychotic phase were excluded because of their inability to give consent. No organic problems were identified in any patients with schizophrenia by medical examinations. All cases and controls completed consent forms and data was collected on each subjected by trained medical students completing two instruments. The demographic questionnaire recorded sex, employment, education, paternal age, maternal age, paternal and maternal age at parturition, family history of psychotic disorders, number of children, birth rank within siblings, and age of disease onset. Both the patients and their carers were asked about the presence of similar disorders in first-degree and second-degree relatives. Those with evidence for positive family history underwent for further evolution by contacting their psychiatrist, inspecting their medical records or structured interview of the subjects and their carers in order to confirm or rule out the presence of mental diseases. The second diagnostic instrument was the Structured Clinical Interview for DSM Disorders (SCID), previously validated for diagnosis of schizophrenia in the Iranian population.

Differences between study groups were examined using the χ^2 ^test for categorical variables, and an independent sample t test (or Mann-Whitney U test) for continuous variables. Probability values of *P *< 0.05 were considered statistically significant. Multiple logistic regression analysis was performed with adjustment for independent association of all factors with schizophrenia as a dependent variable. One model (included all independent variables at one stage) was developed to adjust for covariates. The analysis was performed using SPSS software (V.11.5; SPSS, Chicago, IL, USA).

## Results

A greater number of cases (Table 1) had a birth rank of ≥3 versus controls (35% versus 24%, *P *= 0.01, OD = 1.7, 95% CI 1.12 to 2.5). The difference in the occurrence of psychiatric disorders, including schizophrenia, schizoaffective disorder and major mood disorders (unipolar and bipolar disorder) in first-degree relatives was significant in case versus control groups (*P *= 0.04): 20.5% versus 13.2%, respectively (Table 1).

**Table 1:** Demographic characteristics of cases and controls

	Cases (%)	Controls (%)	*P *value
Sex:			

Male	62.7	61.8	0.84^a^

Female	37.3	38.2	

Education:			

Illiterate	3.6	5	0.87^a^

Lower than diploma level	48.2	49.5	

Diploma	39.5	35.5	

Higher than diploma	86	10	

Paternal age at parturition (mean ± SD)	30.5 ± 6.2	26.4 ± 5.6	0.001^b^

Maternal age at parturition (mean ± SD)	25 ± 4.5	26.1 ± 5.4	0.02^c^

Familial history of major psychiatric disorder:			

Yes	45(20.5%)	29(13.2%)	0.041^a^

No	155(79.5%)	171(86.8%)	

Birth rank			

1	48 (21.8)	61 (27.7)	0.037^b^

2	95 (43.2)	106 (48.2)	

≥3	77 (35%)	53 (24.1)	

*P *values < 0.05 were considered statistically significant.

^a^χ^2 ^test; ^b^Mann-Whitney U test; ^c^t test.

**Figure 1:**
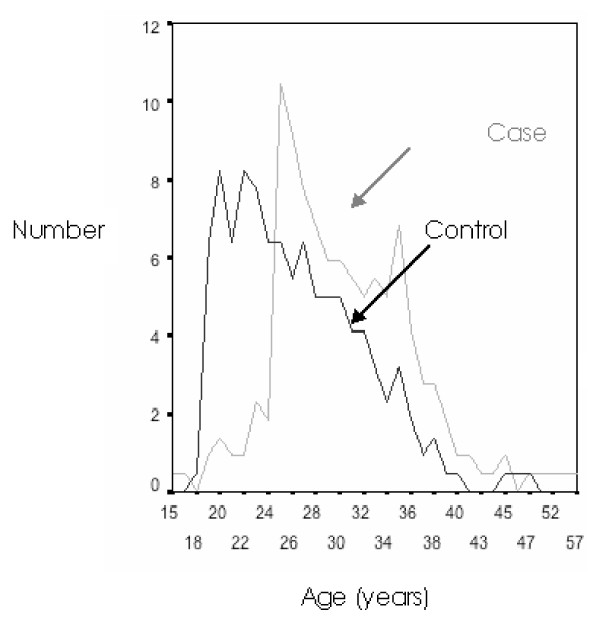
**Distribution of paternal age in case and control subjects**.

Paternal age at parturition of the proband was significantly different between the case and control groups. For instance, the mean paternal age at birth was 30 ± 6.26 versus 26.45 ± 5.64, in case versus control groups, respectively (*P *= 0.0001) (Tables 1 and 2). A significantly higher number of fathers with an age of ≥32 were in the case group versus the control group (Table 3). Analysis of the paternal age distribution (Figure 1) revealed that the fathers of the case group tended to have children at an earlier age, but that a greater number (76, 35%) of cases' fathers age were ≥32, than those in control group (n = 33, 15%). Individuals with fathers' ages at birth ≥32 were 2.77 times at higher risk for schizophrenia (*P *< 0.0001), OR = 3.7 (95% CI 1.9 to 7.3). The mean age of mothers at birth was marginally different (*P *= 0.02) between the case and control groups (26 versus 25.07, respectively). Logistic regression analysis (Table 4), adjusting for the effect of maternal age, birth rank and family history, indicated that paternal age is an independent predisposing factor to schizophrenia; its OR was 3.78 (95% CI 1.9 to 7.3).

**Table 2:** Analysis of the effect of paternal age on cases (n = 220) and controls (n = 220)

		Estimate	Standard error	Wald	Degrees of freedom	Significance	95% CI	
							
							Lower bound	Upper bound
Family history		0.458	0.268	2.921	1	0.087	0.387	1.702

Birth rank	01 sFebruary	0.281	0.355	0.626	1	0.429	0.984	0.984
	
	3.3+	0.286	0.317	0.817	1	366	0.415	0.415

Paternal age	32+	1.425	0.39	13.336	1	0	0.334	0.334

Maternal age	32+	-0.258	0.421	0.376	1	0.54	2.189	2.189

**Table 3:** Sample classes as a function of parental or maternal age at birth

Group	Parental age at birth, n (%)				Total (*P *= 0.065)
		
	Paternal		Maternal		
		
	<32 years	>32 years	<32 years	>32 years	
Cases	144 (76)	76 (34.5)	195 (88.5)	25 (11.5)	220 (100)

Controls	187 (85)	33 (15)	206 (93.5)	14 (6.5)	220 (100)

**Table 4:** Stepwise analysis of the effect of paternal age on cases, controls

		B	Standard error	Significance	Exp(B)	95% CI for Exp(B)	
						
						Lower	Upper
Step 1	Birth rank	0.313	0.131	0.017	1.368	1.058	1.769
	
	Constant	-0.642	0.285	0.025	0.526		

Step 2	Birth rank	0.237	0.142	0.096	1.268	0.959	1.676
	
	Family history	0.481	0.263	0.067	1.618	0.967	2.707
	
	Maternal age	0.387	0.377	0.304	1.473	0.704	3.083
	
	Constant	-0.599	0.297	0.044	0.550		

Step 3	Birth rank	-0.115	0.169	0.496	0.891	0.640	1.241
	
	Family history	0.444	0.268	0.098	1.559	0.922	2.638
	
	Maternal age	-0.374	0.431	0.386	0.688	0.296	1.602
	
	Paternal age	1.329	0.336	0.000	3.778	1.955	7.304
	
	Constant	-0.123	0.321	0.703	0.885		

This case-control study providing data on a previously unexplored Iranian population supports the hypothesis that advanced paternal age and a birth rank of higher than 3 (that is, higher age of father at birth) are linked to schizophrenia pathogenesis.

## Discussion

Our results linking advanced paternal age to schizophrenia are comparable to observations in other populations. Further, logistic regression analysis has suggested that higher birth rank along with advanced paternal age is more of a risk factor for schizophrenia than maternal age. Birth rank and short birth interval were previously found to be risk factors for schizophrenia [30-32]. The effect of higher birth order/rank may reflect the costs of childbearing on a mother's long-term health, as Christensen *et al. *analysing twin data found support for the proverb 'a tooth, a child' [33]. Higher birth rank effects may also be a function of advanced paternal and/or maternal age. Along these lines, our study of large families provides evidence for the first time that the relationship between birth order/rank and schizophrenia may be due to paternal age. In fact, as advanced paternal age was a risk factor for schizophrenia, the disease frequency was greater in children with higher birth rank as well (that is, higher paternal age). Higher birth rank (and advanced paternal age) may account for the coincidence of disease in multiple siblings, whereas such coincidences are taken as evidence for genetic inheritance. However, paternal age effects on schizophrenia pathogenesis may be mediated through both genetic and/or epigenetic mechanisms [34,35].

Epigenetics refers to heritable but potentially reversible changes in DNA methylation, RNA (editing and interference), and protein (for example, histone) modification and provide mechanisms for the environment to interact with the genome [36]. The multiplicity of targets and modifications linked to epigenetic programming suggests that perturbations to such pathways are likely to account for a large amount of phenotypic variations. Advanced paternal age is associated with an elevated incidence of environmental exposures that may affect the spermatozoid epigenetic memory. These epigenetic changes could be the underlying mechanisms of paternal age effects on schizophrenia pathogenesis.

About 50% of monozygotic (aka identical) twins afflicted by disease and starting life with identical genomes are discordant for schizophrenia, and when concordant they do not have the same psychiatric phenotypes [1]. Additionally, all children of monozygotic twins concordant or discordant for schizophrenia have the same elevated probability of becoming ill [37,38]. These observations suggest that genetic susceptibility is transmitted to offspring, but environmental factors also contribute in disease occurrence and presentation. An elevated somatic mutation rate in twins discordant for schizophrenia was detected around simple trinucleotide repeat sequences, for example, (CAG)_n _[39]. It is of note that this study was limited to (CAG)_n _repeat occurrences, and we do not know whether the increased mutation rate extends to other sequences. However, other studies have also detected an increase in neuronal aneuploidies in schizophrenic brains [40,41]. Several groups have suggested that the genetic-environmental linkage reflects the need for 'two-hits' (genetic plus somatic changes) to DNA. Other studies have shown that genetic and epigenetic changes occur with age [42,43], and epigenetic differences exist between monozygotic twins [44,45].

In sperm, dominant and codominant base change mutations will impact progeny and subsequent generations in a simple Mendelian fashion. A recessive mutation will not be detected in progeny (except occasionally when a second mutated allele is present) unless the target gene is maternally imprinted and silenced. Epigenetic changes at imprinted genes could lead to inappropriate expression or lack of expression. The effects on subsequent generations will be dependent on the parental origin of the change. Further, epigenetic DNA methylation is reset during passage through the germline and during embryonic and post natal development; hence, it is not clear what changes will be passed to subsequent generations [36].

The dissection of the paternal age effect in large families is advantageous given the plethora of potential DNA changes and the consequences for subsequent generations. The paternal age effect may be due to inadequate nutrition and/or exposure to environmental toxins during spermatogenesis. For instance, it is estimated that 30% of American diets are deficient in folate [46,47], a nutrient required for the biosynthesis of TTP and DNA synthesis as well as purine nucleotides that are necessary for DNA and RNA synthesis and other cellular processes (for example, epigenetic changes to RNA and proteins, energy transduction, and signally). The success of genetic studies on rare human disease has prompted research on complex common disease to focus on genetic approaches while largely ignoring environmental factors that have the potential to change not only DNA but also cause epigenetic changes to DNA, RNA and proteins. For instance, paternal age and occupations linked to schizophrenia in progeny may involve exposures that induce oxidative stress and impact folate metabolism [48]. A comprehensive understanding of schizophrenia and other common complex diseases requires studies on the interaction of the environment with specific genotypes, some predisposed, to severe illness. Such studies are required as several preventable known environmental and nutritional factors (for example, folic acid deficiency) can induce epigenetic alterations that may impact the quality of human life in general.

## Competing interests

The authors declare that they have no competing interests.

## Authors' contributions

H-RA designed the study, collected clinical sample data and drafted the manuscript. BM participated in sample collection. MN performed data analysis and CLS contributed to the editing and writing of the manuscript. All authors read and approved the final manuscript.
